# Vertebrobasilar dolichoectasia compresses midbrain, worsens Parkinson symptoms, and evokes Holmes tremor: A case report

**DOI:** 10.1097/MD.0000000000047275

**Published:** 2026-01-16

**Authors:** Jian Li, Nan Cheng, Tihua Liu, Qinfeng Rong

**Affiliations:** a Department of Neurology, The People’s Hospital of Huantai Country (The 9th People’s Hospital of Zibo), Zibo, Shandong Province, China.

**Keywords:** Holmes tremor, motor symptoms, nonmotor symptoms, Parkinson disease, symptomatic treatment, vertebrobasilar dolichoectasia

## Abstract

**Rationale:**

Parkinson disease (PD) is characterized by bradykinesia, resting tremor, rigidity, as well as other motor and non-motor symptoms due to damage to dopaminergic neurons in the substantia nigra. Holmes tremor (HT), otherwise, manifests itself as resting, intentional, and postural tremors in the limbs, resulting from lesions in the red nucleus of the midbrain. These 2 distinctive maladies seldom emerge simultaneously. This study delineates a case with both PD and HT occurring after vertebrobasilar dolichoectasia.

**Patient concerns:**

A 54-year-old male patient experienced a gradual aggregation of static and kinetic postural tremors in the last 2 years.

**Diagnosis:**

Brain magnetic resonance imaging revealed vertebrobasilar dolichoectasia compressing the midbrain, whereas intracranial ultrasound confirmed the diagnosis of PD.

**Interventions:**

The patient received long-term medication with levodopa and benserazide hydrochloride tablets, pramipexole hydrochloride, and trihexyphenidyl.

**Outcomes:**

The tremor was resolved, and the quality of life was improved substantially.

**Lessons:**

In patients with both PD and HT, the identification of underlying causes and the proper symptomatic treatment are crucial for relieving the symptoms. However, the ultimate approaches would be microvascular decompression, deep-brain stimulation, or stereotactic thalamotomy.

## 1. Introduction

Parkinson disease (PD) manifests itself as a chronic, progressive, and degenerative disease involving midbrain substantia nigra and striatum, which predominantly affects the elderly.^[1]^ Motor symptoms (MS) such as static tremor, bradykinesia, rigidity, postural and gait anomaly typify the main features of PD.^[1]^ Moreover, various nonMS (NMS), such as hyposmia, constipation, depression, and sleep disorders, may also appear. As the disease progresses, both MS and NMS worsen quickly. Motor complications often show up in the later stages, ultimately leading to long-term bed rest and worsening quality of life.^[1]^ The incidence of PD has been increasing since 1980s, coinciding with the aging of the population.^[2]^ Different from PD, Holmes tremor (HT) is characterized by resting, intentional, and postural tremors in the limbs, resulting from lesions in the red nucleus of the midbrain. Thus far, except for a few prospective studies or case reports, there is no large-scale clinical research on HT.^[3]^ As both PD and HT are associated with the midbrain, if any complication perturbs the area, it may provoke or aggravate the 2 disorders.

Vertebrobasilar dolichoectasia (VBD) represents a type of intracranial vascular anomaly with unclear mechanisms. The primary features comprise dilation, elongation, and twisting of basilar and vertebral arteries. Multiple neurological events may be caused by VBD, such as ischemic and hemorrhagic lesions of posterior circulation, cranial nerve compression, and obstructive hydrocephalus, which increase mortality and exacerbate disability of patients. Thus far, targeted guidelines for the treatment of VBD are still lacking.^[4]^

Herein, we reported a unique case with concurrent VBD and PD, with the patient exhibiting both static and kinetic postural tremors (HT). The patient received a quick diagnosis, and his medications were adjusted accordingly. All the countermeasures prevented further decline of his condition, creating an opportunity for further operations.

## 2. Case presentation

**Figure 1.**
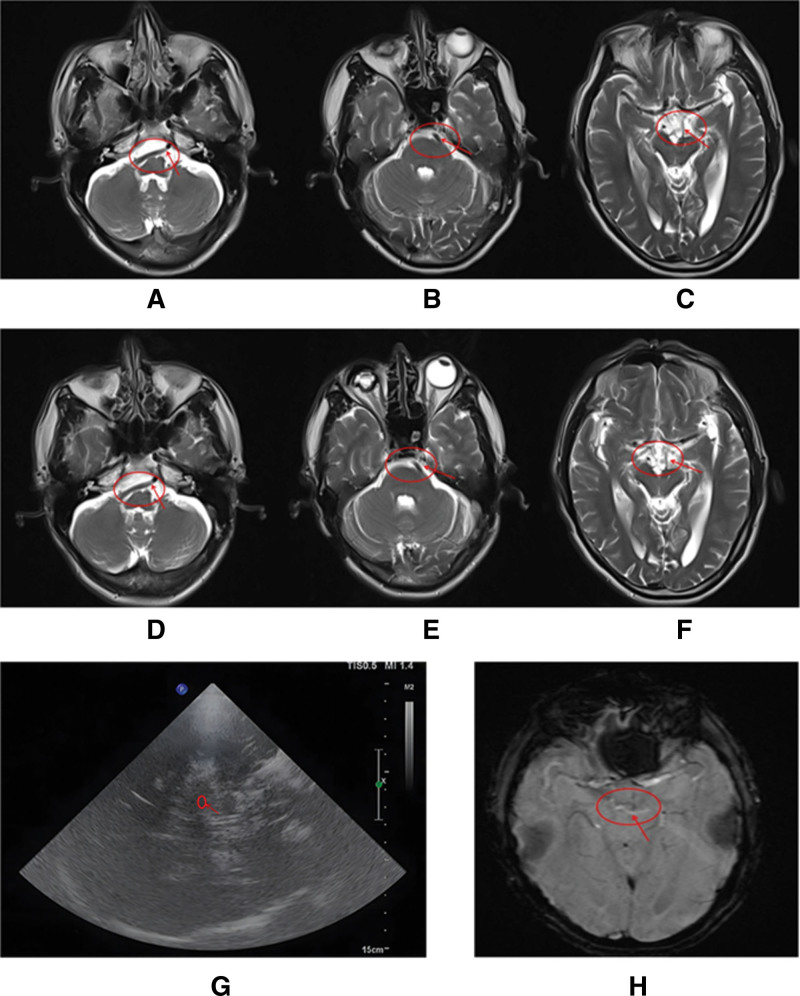
Brain MRI of the patient in 2022 (A–C) and 2024 (D–F) showed a dilation and elongation of basilar artery, which compressed the left midbrain. The severity of tremors was higher in 2024 than in 2022. Cranial ultrasound examination showed strong patchy echoes in substantia nigra (III grade) with an area of >0.2 cm^2^ (G). Susceptibility-weighted imaging also found the dilation and elongation of basilar artery, which compressed both sides of the midbrain (H). Red circles and arrows indicate the lesion sites. MRI = magnetic resonance imaging.

**Figure 2.**
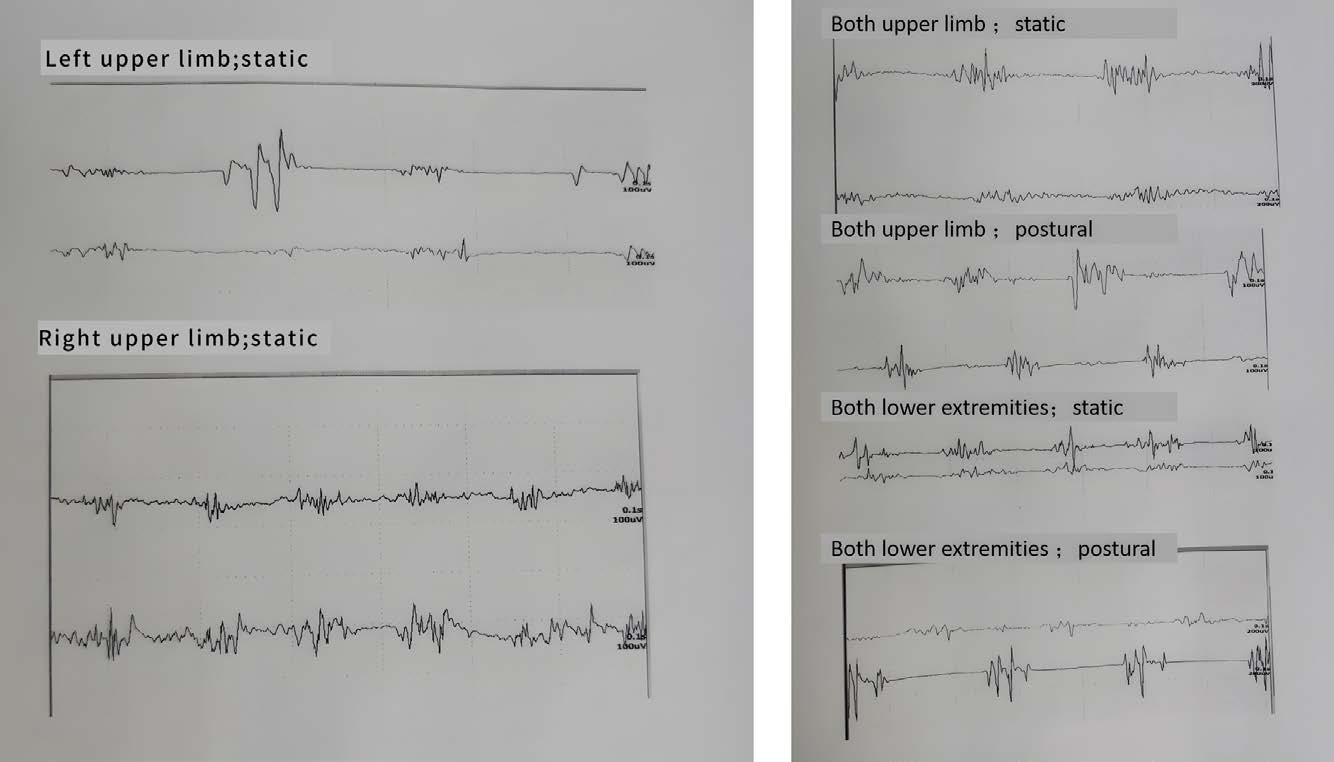
Tremor analysis in 2022 (A) showed a synchronously static tremor with a peak frequency between 4.5 and 5.5 Hz and amplitude ranging from 30 to 193 µV in the upper limbs. Tremor analysis in 2024 (B) revealed a synchronously static tremor with a frequency of 3 to 5 Hz and an amplitude of 20 to 197 µV, and an alternating postural tremor with a frequency of 2.7 to 3.5 Hz and an amplitude of 43 to 360 µV in the upper limbs. Furthermore, it revealed a synchronously static tremor with a frequency of 3.2 to 5 Hz and an amplitude of 45 to 218 µV, and an alternating postural tremor with a frequency of 3 to 4.8 Hz and amplitude of 87 to 315 µV in the lower limbs.

A 54-year-old male patient visited our department on June 8, 2022, who exhibited a forward-leaning posture during walking, with slow movement, static tremor in the left hand, difficulty with fine motor skills like getting out of bed, turning over, and buttoning clothes. These symptoms first presented in October 2021, worsened gradually, and remained untreated till this visit. We noticed a static tremor in both hands, with the left-hand tremor being more pronounced. Neurological exam revealed no abnormality in the response of cranial nerves. In all limbs, the scores of manual muscle test (MMT-8) were 5 and the muscle tensions were high. Brain magnetic resonance imaging (MRI) discovered a dilated and elongated basilar artery, which compressed the left midbrain (Fig. 1A–C). Tremor analysis (both upper limbs) noticed a synchronously static but not postural tremor, with a peak frequency of 4.5 to 5.5 Hz and an amplitude of 30 to 193 µV (Fig. 2A). Cranial ultrasound examination detected strong patchy echoes in the substantia nigra (Grade III), covering an area larger than 0.2 cm² (Fig. 1G). The patient’s symptoms, tremor analysis, and ultrasound revelation supported the diagnosis of PD, and thus, levodopa and benserazide hydrochloride tablets (0.125 g, po tid, China SFDA approval number: H10930198, manufacturer: Shanghai Roche Pharmaceutical Co., Ltd.) was administered. Six days later, the patient was discharged with PD symptoms partially alleviated. Since then, until January 2024, the patient remained in a stable condition.

By February 2024, the symptoms worsened again, presenting as severe involuntary tremors and affecting all 4 limbs. The tremors intensified in a sitting position and eased slightly in a standing position. His daily activities were hampered and reactions became slower. On July 19, 2024, he revisited our department for a consultation. The neurological tests were normal. Muscle force in all limbs was rated at 5 with higher tension relative to the previous check. Severe static and kinetic tremors were noted in all limbs, with a lower frequency than before. Sensory examination showed no abnormalities, but coordination tests could not be completed because of intense tremors. A marked dilation and elongation of the basilar artery was identified on brain MRI, severely compressing the left midbrain (Fig. 1D–F). Susceptibility-weighted imaging also found the dilation and elongation of the basilar artery, which compressed both sides of the midbrain (Fig. 1H). Tremor analysis of the upper limbs revealed a synchronously static tremor with a frequency of 3 to 5 Hz and an amplitude of 20 to 197 µV, and an alternating postural tremor with a frequency of 2.7 to 3.5 Hz and an amplitude of 43 to 360 µV. Tremor analysis of lower limbs detected a synchronously static tremor with a frequency of 3.2 to 5 Hz and an amplitude of 45 to 218 µV, and an alternating postural tremor with a frequency of 3 to 4.8 Hz and an amplitude of 87 to 315 µV (Fig. 2B). The PD scale score was higher in 2024 than in 2022. Based on the worsened symptoms and concurrent severe static and kinetic tremors in all limbs, particularly upon movement, the presence of PD and HT intensified by VBD was deduced without much challenge. Then, the medication was adjusted as levodopa and benserazide hydrochloride tablets (0.125 g, po tid, qn), pramipexole hydrochloride (0.125 mg po tid), and trihexyphenidyl (1 mg po tid). The patient underwent the treatment with favorable adherence and tolerability. After 6 days of medication, the tremors in all limbs were alleviated, with similar intensity noted in sitting and standing positions. During the 1-month follow-up visit, his right hand showed no signs of tremor. Meanwhile, the daily life returned normal without any adverse and unanticipated event. Thus, the medication was continued without any modifications. Nevertheless, if possible, microvascular decompression of cranial nerves along with deep brain stimulation or stereotactic thalamotomy will be considered when the efficacy of the current treatment shows any decline.

## 3. Discussion

This case typified a rare condition wherein HT occurred concurrently with PD because of VBD compressing the midbrain. The patient was diagnosed in time, and the medication was adjusted in line with his diagnosis; these changes improved the patient’s quality of life greatly.

The exact cause of VBD remains unclear, and thus, the diagnosis mainly relies on imaging techniques.^[5]^ In previous studies, VBD has been reported to evoke ischemic or hemorrhagic lesions in the supplied areas, as well as compression or stimulation of nearby cranial nerves, brainstem, or the third ventricle. Among these regions, the brainstem shows higher tolerance.^[6]^ However, upon severe compression, symptoms like hearing loss, tinnitus, and dizziness may arise due to the damage of the vestibulocochlear nerve. VBD may compress all cranial nerves, particularly V, VII, and VIII, and thus, the main clinical manifestations include 1-side facial muscle spasms, trigeminal neuralgia, hoarseness, and dysphagia. Nystagmus and ocular muscle paralysis are uncommon. Usually, the compression affects just 1 cranial nerve; however, with severe elongation and tortuosity of the vertebrobasilar artery, multiple nerves may be affected, leading to a complicated situation. However, it is very rare for VBD to exacerbate Parkinson symptoms. In this case, the PD first affected the left side of the body and subsequently perturbed the right side. The patient’s condition declined rapidly, exhibiting bilateral static and kinetic postural tremors after 2 years. Based on MRI scans, cranial ultrasound, and tremor analysis, the main cause of the manifestation was inferred to be the twisted basilar artery which compressed both sides of the midbrain.

HTs have been described as resting, intentional, and postural tremors, which typically disturb proximal muscles of limbs with a lower frequency (<4.5 Hz). It usually occurs 2 weeks to 2 years after the onset of the disease with a lesion in the midbrain, for which dopamine or dopamine receptor agonists seem effective.^[7]^ The red nucleus serves as a relay station for various conduction pathways in the brain, the damage of which may cause tremors in the corresponding body areas.^[8]^ In this case, the patient’s symptoms conformed to HT, and the underlying mechanism involved lesions on the red nucleus that simultaneously damaged the substantia nigra–striatal and cerebellar–thalamic–cortical/dentate nucleus–red nucleus–olive pathways.^[9]^

Thus far, HT has typically been treated using medication and surgical intervention. Levodopa, dopamine receptor agonists, clonazepam, anticholinergic, and antiepileptic drugs are recommended.^[10]^ Among these, levodopa has been found to be particularly helpful. Anticholinergic drugs and clonazepam can also alleviate the severity of the condition. However, clonazepam’s sedative effect may compromise the patient’s working ability, and high doses of anticholinergic drugs cause adverse effects as well. In our case, the patient was a working middle-aged male, and thus, surgical treatment was not the first choice. The medication scheme was based on levodopa, dopamine receptor agonists and a small dose of anticholinergic drugs. This approach worked promptly with minimal adverse effects, which provides a reference for the management of similar cases. Furthermore, our case highlights the importance of recognizing and addressing rare situations in PD patients with rapidly progressing clinical symptoms.

Taken together, for patients having both PD and HT, the proper symptomatic treatment is crucial for relieving the condition. However, if possible, the ultimate treatment remains a combination of microvascular decompression, deep brain stimulation or stereotactic thalamotomy.

## Acknowledgments

We express our gratitude to Medlive Inc. for assisting us with this manuscript.

## Author contributions

**Conceptualization:** Jian Li, Nan Cheng, Tihua Liu, Qinfeng Rong.

**Formal analysis:** Jian Li, Tihua Liu.

**Investigation:** Nan Cheng, Qinfeng Rong.

**Project administration:** Nan Cheng.

**Resources:** Tihua Liu.

**Writing – original draft:** Nan Cheng.

**Writing – review & editing:** Jian Li, Tihua Liu, Qinfeng Rong.
